# Enterovirus A71 neurologic complications and long-term sequelae

**DOI:** 10.1186/s12929-019-0552-7

**Published:** 2019-08-08

**Authors:** Luan-Yin Chang, Hsiang-Yuan Lin, Susan Shur-Fen Gau, Chin-Yu Lu, Shao-Hsuan Hsia, Yhu-Chering Huang, Li-Min Huang, Tzou-Yien Lin

**Affiliations:** 1 Departments of Pediatrics, National Taiwan University Children’s Hospital, College of Medicine, National Taiwan University, No. 8, Chung-Shan South Road, Taipei, Taiwan; 2 Psychiatry, National Taiwan University Children’s Hospital, College of Medicine, National Taiwan University, Taipei, Taiwan; 3 Departments of Pediatrics, Chang Gung Children’s Hospital, Chang Gung University, Taoyuan, Taiwan

**Keywords:** Enterovirus A71, Encephalitis, Rhombencephalitis, Polio-like syndrome, Sequelae, Tracheostomy, Cognition, Attention deficiency hyperactivity disorder, Age of onset

## Abstract

During recent 20 years, enterovirus A71 (EV-A71) has emerged as a major concern among pediatric infectious diseases, particularly in the Asia-Pacific region. The clinical manifestations of EV-A71 include uncomplicated hand, foot, and mouth disease, herpanina or febrile illness and central nervous system (CNS) involvement such as aseptic meningitis, myoclonic jerk, polio-like syndrome, encephalitis, encephalomyelitis and cardiopulmonary failure due to severe rhombencephalitis. In follow-up studies of patients with EV-A 71 CNS infection, some still have hypoventilation and need tracheostomy with ventilator support, some have dysphagia and need nasogastric tube or gastrostomy feeding, some have limb weakness/astrophy, cerebellar dysfunction, neurodevelopmental delay, lower cognition, or attention deficiency hyperactivity disorder. Long term sequelae may be related to greater severity of CNS involvement or neuron damage, hypoxia and younger age of onset.

## Introduction

Enterovirus 71 (EV-A71) was first isolated in California, USA in 1969 [1]. Since then, EV-A71 has been detected worldwide [2–7]. Large-scale outbreaks with lots of central nervous system (CNS)-complicated cases and deaths occurred in Bulgaria, Hungary, Malaysia, Taiwan, Vietnam, Brunei, China and Cambodia [2–8]. During recent 20 years, it has emerged as a major concern among pediatric infectious diseases, particularly in the Asia-Pacific region.

A nationwide EV-A71 epidemic occurred in Taiwan in 1998, which caused 405 severe cases and 78 deaths [8–12]. After the epidemic, integrated multiple national enterovirus surveillance systems were established by the Taiwan Centers for Disease Control [13–16]. These systems include viral lab network; outpatient, inpatient, and emergency room visits for hand-foot-and-mouth-disease (HFMD) and/or herpangina (HA) from National Health Insurance claims data; mandatory notification of enterovirus severe cases, in which throat swab, serum, and contact information is collected through an epidemiological investigation. After the first EV-A71 epidemic in 1998 in Taiwan, nationwide epidemics occurred again in 2000–2001, 2005, 2008, and 2012 based on the surveillance data [15–17]. A stage-based management was developed for the treatment of EV-A71 infection in 2000, and it lowered the case-fatality rate of severe EV-A71 cases [11, 18] but long-term sequelae are very concerning, especially in young children.

Neurodevelopment and cognitive function may be affected by viral encephalitis or by bacterial meningitis [19–24]. The fact that the survival rate of children with EV-A71 CNS infections has improved after a stage-based management, shows that it is important to monitor neurological and functional outcomes. In this review, the authors will review clinical manifestations, neurological complications and long-term sequelae of EV-A71 infections.

### Uncomplicated clinical manifestations

Although EV-A71 can infect both adults and children, their clinical outcomes diverge a lot. From our household EV-A71 study [25], we found that only 6% of 183 infected children, were asymptomatic, 73% had uncomplicated illnesses of hand, foot and mouth disease, herpangina or nonspecific febrile illness and 21% suffered from complications of central nervous system involvement and/or cardiopulmonary failure. On the contrary, 53% of 87 infected adults were asymptomatic, 39% had nonspecific illnesses with fever, sore throat or gastrointestinal discomfort and only 8% (7/87) had hand, foot and mouth disease. All symptomatic adults recovered completely from uncomplicated illnesses in our household transmission study [25]. However, adult-onset encephalitis caused by intrafamilial transmission of EV-A71 was ever reported in Japan and it elucidated the risk for EV-A71 encephalitis even in adults [26].

Interestingly, in a seroepidemiologic study [27], only 29% of the preschool children infected with EV-A71 developed HFMD/HA, indicating about 70% of community-acquired infected children might be asymptomatic. Therefore, household transmission produced a higher rate of clinical symptoms (94%) than extra-household transmission (29%). Viral load or host genetic factors may account for this difference but this hypothesis will need further confirmation. However, we can not exclude that recall bias from retrospective nature of the seroepidemiologic study [27] may underestimate the true rate of clinical symptoms for extra-household transmission.

According to previous clinical studies [10, 11, 18, 28–30], symptomatic EV-A71 infection can progress through four stages: HFMD/herpangina (Stage 1), CNS involvement (Stage 2), autonomic nervous system dysregulation and/or cardiopulmonary failure (Stage 3), and convalescence (Stage 4). Most EV-A71 cases in those studies stayed at Stage 1, some progressed to Stage 2 and a few would advance to the most severe condition, Stage 3 (Table 1). The Stage 4 is the convalescent stage, including long-term sequelae needing further rehabilitation and medical care.

**Table 1:** Clinical manifestations of EV-A71 infection at different stages

Stage	Diagnosis	Manifestations
1	HFMD, herpangina, fever	Fever, oral ulceration, vesicular rash or small erythematous maculopapular rash on the hands, the feet, the knees, or the buttocks
2	CNS involvement including aseptic meningitis, myoclonic jerk, encephalitis, polio-like syndrome, encephalomyelitis	Myoclonic jerk, limb weakness, lethargy, headache, vomiting, upward gaze, nystagmus, wandering eyes, seizure
3	Autonomic nervous system dysregulation and/or cardiopulmonary failure after CNS involvement	Tachycardia, hyperthermia, profuse sweating, transient hypertension, tachypnea, hypoxia, shock,
4	Convalescence	Some with complete recovery but some with long-term sequelae shown in Table 2

*HFMD* hand, foot, and mouth disease, *CNS* central nervous system

The uncomplicated EV-A71 illnesses include HFMD, herpangina, pharyngitis, nonspecific febrile illness, generalized viral exanthema, and enteritis. The cases may have fever for 1 to 3 days, reaching sometimes higher than 39 degrees. HFMD patients have oral ulcers on the tongue and buccal mucosa, plus vesicular rash or small erythematous maculopapular rash on the hands, the feet, the knees, or the buttocks. The EV-A71-induced vesicular or maculopapular rash over the extremities is sometimes so tiny that it may be overlooked by parents and even doctors. Herpangina includes oral ulceration on anterior tonsillar pillars, the soft palate, buccal mucosa or the uvula. Oral ulceration causes pain while eating or drinking and patients may need intravenous fluid supplement if dehydration occurs. About 10 to 20% of the EV-A71 cases have febrile illness or pharyngitis without the usual HFMD/herpangina [25].

### Complicated EV-A71 illness with CNS involvement

EV-A71 infection may develop complicated conditions 1 to 5 days after the onset of the illness. After suffering the initial HFMD, herpangina or febrile illness and an intermittent fever that usually lasts from 3 to 7 days, some patients may begin to suffer mild CNS involvement such as myoclonic jerk and aseptic meningitis, or severe CNS involvement such as encephalitis, polio-like syndrome or encephalomyelitis, which are Stage 2 in our clinical classification (Table 1). The most severe complication, Stage 3 in our clinical classification (Table 1), is autonomic nervous sytem dysregulation, cardiopulmonary failure or neurogenic pulmonary edema following severe brainstem encephalitis (rhombencephalitis) [30]. The risk of neurologic complications may be associated with younger age, male gender and some host genetic factors [10].

EV-A71 cases with aseptic meningitis usually have myoclonic jerks during sleep, vomiting, headache or irritable crying. They have no or only mild neck stiffness. Aseptic meningitis usually recovers 3 to 7 days after hospitalization.

The most common initial symptoms of EV-A71 encephalitis are myoclonic jerks during sleep and followed by other symptoms or signs of encephalitis. Patients with EV-A71 encephalitis may show signs of changes in consciousness such as lethargy, sleepiness, seizure, ataxia, cranial nerve palsy, such as abducens palsy, facial palsy, dysphagia, upward gaze, nystagmus and wandering eyes [30]. Patients may also show subtle symptoms of increased sympathetic tone, such as insomnia, profuse sweating, paralytic ileus, neurogenic bladder, panic or increased startle reflex. If cases of encephalitis do not advance to Stage 3, they usually recover without sequelae 5 to 10 days later.

EV-A71 cases with poliomyelitis-like syndrome have asymmetric acute limb weakness plus decreased reflex and usually show no disturbance of limb sensation. For example, the affected children suddenly cannot walk or raise their arms, or they easily fall down 3 to 7 days after HFMD or herpangina. About one half of EV-A71 polio-like cases have had long-term sequelae of limb weakness and atrophy. Cases of encephalomyelitis have symptoms of both encephalitis and poliomyelitis-like syndrome.

Brain or spinal computer tomography (CT) usually yields negative findings in cases with EV-A71 CNS infection and are thus not the image study of choice. Magnetic resonance imaging (MRI) is a better option, as MRI studies usually show hyperintensity in the CNS lesions on T2-weighted images [30]. The major CNS lesions of brainstem encephalitis and cerebellitis are in the medulla oblongata, pons, midbrain, and the dentate nuclei of the cerebellum [10, 29, 30]. For the polio-like syndrome, the lesions involve the anterior horn of the spinal cord. Some might have normal MRI results. Cases of encephalomyelitis might have lesions in both brainstem and spinal cord. In the follow-up MRI examinations of patients with neurological sequelae, the brain lesions may persist 1 to 3 years after their acute episode of the illness.

Although the clinical manifestations, CSF pleocytosis and image studies all show evidence of CNS involvement, EV-A71 is seldom isolated from a patient’s cerebrospinal fluid.

### Autonomic nervous system dysregulation and/or cardiopulmonary failure following CNS involvement

Several hours to 2 days after the onset of CNS involvement, some patients may advance into Stage 3 at which time they have sudden signs of tachypnea, tachycardia (135–250 beats per minute), transient hypertension, profuse sweating, cyanosis and profound shock. Patients are usually alert except for mild lethargy and are sometimes found to have transient hypertension in the beginning of Stage 3. Significant laboratory findings include hyperglycemia and leukocytosis. Chest X-ray films show alveolar density and no cardiomegaly. Specifically, most patients during the 1998 epidemic had complete white-out on chest X-ray within 12 h entering into Stage 3 [10]. Their electrocardiographic examination shows sinus tachycardia and no arrhythmia. Ejection fractions on cardiac echography are about 40 to 80%. Once intubated, children produce a white frothy secretion, followed by a pink frothy fluid and then fresh blood from endotracheal tube. Patients frequently have persistent fever and profuse sweating during critical points of pulmonary edema/hemorrhage [10].

Progressive hypotension or shock, oliguria or anuria, tachycardia and decreased levels of consciousness are identified if the disease keeps progressing [10]. Nearly 80% of these children during 1998 epidemic died within 12 h of intubation, but the fatality rate had decreased to 30–40% from 2000 to 2002 [18]. The significantly lower mortality rate may arise from the intensive care with earlier start and better quality, because a stage-based management has been developed since 2000 [11, 18]. In recent 10 years, the case-fatality rate in Taiwan has been even lower to less than 10% [17], and it may be related to early precaution of severe cases, better intensive care and advanced life support such as extra-corporeal membrane oxygenation.

## Long-term sequelae

The stage-based management was developed for the treatment of EV-A71 infection [11] and it has lowered the case-fatality rate of severe EV-A71 cases significantly [18], but long-term sequelae are of great concerns, especially in young children. Overall, long-term sequelae are related to greater clinical severity at the acute stage, neuronal damage, hypoxia and younger age of onset. Patients with mild CNS involvement such as myoclonic jerk or aseptic meningitis would recover completely without neurologic sequelae [31]. Children with brainstem encephalitis plus cardiopulmonary failure have the highest rate of sequelae and most severe multiple sequelae in general [31–33]. The long-term sequelae include dysfunctional aerodigestive tract, neurological sequelae, delayed neurodevelopment, impaired cognition, as well as psychosocial problems (Table 2).

**Table 2:** Long-term sequelae of severe EV-A 71 infections

Sequelae	Possible causes
Dysphagia with nasogastric tube or gastrostomy feeding	Neuronal damage of the brainstem
Central hypoventilation with tracheostomy and ventilator support as Fig. 1 shows	Neuronal damage of the brainstem
Limb weakness/atrophy as Fig. 3 shows	Motor neuron damage
Seizure	Neuronal damage or hypoxia
Neurodevelopmental delay	Neuronal damage, younger age of onset or hypoxia
Lower cognition	Neuronal damage, younger age of onset or hypoxia
Behavioral problems, such as ADHD or oppositional symptoms	Cause to be determined

*ADHD* attention-deficit hyperactivity disorder

## Aerodigestive tract and neurological sequelae

**Fig. 1:**
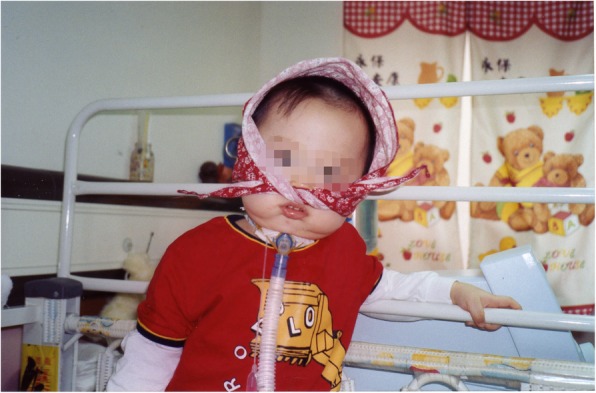
A child who had tracheostomy with ventilator support because he had central hypoventilation

**Fig. 2:**
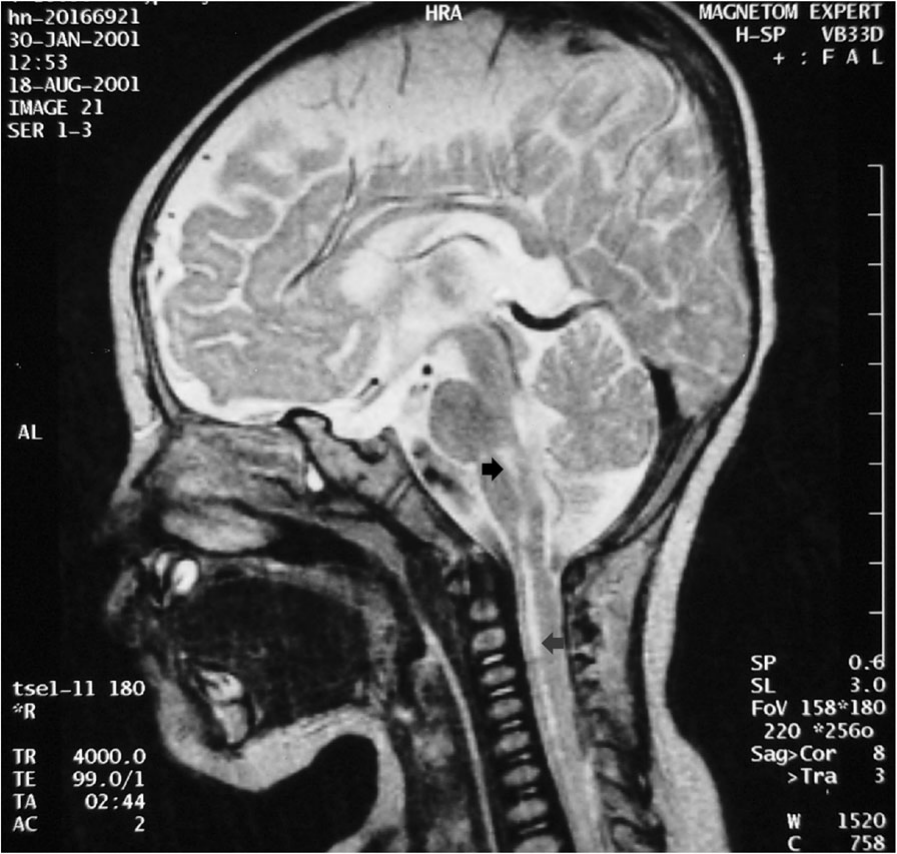
High-intensity lesions in the tegmentum of the brain stem (black arrow) and high-intensity lesions in the cervical spinal cord (gray arrow) on the T_2_-weighted image of MRI in a case with the sequelae of central hypoventilation and right upper limb weakness and atrophy

In our follow-up study (1 to 7 years after the EV-A71 infection), of the 28 patients who had cardiopulmonary failure after CNS involvement, third-fourths had sequelae, including limb weakness and atrophy, facial nerve palsy, dysphagia with tube feeding, central hypoventilation with ventilator support, seizure and psychomotor retardation from hypoxia [31]. Figure 1 shows a child who had tracheostomy with ventilator support because he had central hypoventilation. Patients with sequelae usually had abnormal findings on MRI, including high-intensity lesions in the tegmentum of the brain stem and/or high-intensity lesions in the spinal cord on the T_2_-weighted image (Fig. 2). Among patients who had cardiopulmonary failure after CNS involvement, the percentage with sequelae is significantly higher than that among patients with CNS involvement alone (*P* < 0.001) [31].

Tsou and colleagues [33] followed up patients at least 3 years after discharge from hospitalization for the EV-A71 infection. They found that among 72 patients requiring endotracheal intubation due to respiratory failure or ventilator dependence during acute stage, 14 (19%) underwent tracheostomy, and 10 (14%) underwent gastrostomy. Significant tracheostomy and gastrostomy predictors are age of onset < 2 years, pulmonary edema or hemorrhage, hypotension, hemiparesis, and positive MRI findings. The cause of aerodigestive tract sequelae is most related to neuronal damage of the brainstem as identified by MRI (Fig. 2).

Huang et al. followed up patients with brainstem encephalitis plus cardiopulmonary failure, and found that they had residual neurologic dysfunction, varying from subtle monoparesis to severe bulbar dysfunction, central and peripheral respiratory failure, and flaccid quadriparesis [32]. Some patients also had residual cerebellar defects [32].

**Fig. 3:**
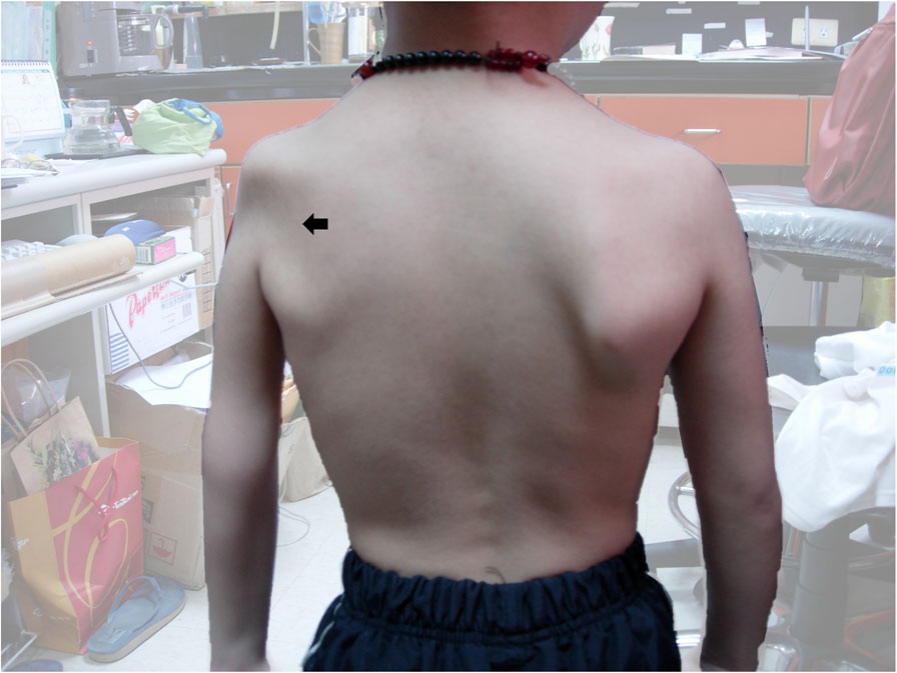
A boy who had left shoulder subluxation and left arm atrophy (arrow) due to polio-like syndrome

For patients with polio-like syndrome, about 50% have limb weakness and atrophy. In our follow-up study, about one half (56%) of patients with a poliomyelitis-like syndrome had unilateral limb weakness and atrophy [31]. Among them, most patients needed limb rehabilitation. Further, a few of them even needed reconstructive surgical interventions. Figure 3 shows a boy who had left shoulder subluxation and left arm atrophy due to the polio-like syndrome.

## Neurodevelopment, cognition and psychosocial outcome

For neurodevelopment outcome from our follow-up study [31], only 1 patient, who had severe CNS involvement without cardiopulmonary failure, had a delay in the gross motor and personal–social categories. Of the patients who had cardiopulmonary failure after CNS involvement, third-fourths were found to have delayed neurodevelopment according to the Denver Developmental Screening Test, 2nd Edition. The clinical severity of the CNS involvement is significantly associated with the children’s neurodevelopment [31].

For cognitive function, the clinical severity and the age at disease onset are significantly associated with IQ scores. For example, the mean full-scale IQ of patients with cardiopulmonary failure after CNS involvement is significantly lower than that of the other patients. Children who were less than 2 years of age at disease onset would have lower full-scale IQs, and are more likely to have a full-scale IQ < 85 than those whose age at onset was 2 years or older [31].

To the best of knowledge, we were the first to conduct a prospective study to follow up long-term behavioral outcomes or attention-deficit hyperactivity disorder (ADHD)-related symptoms in children after an EV-A71 CNS infection [34]. 3–7 years after infection, children who have had EV-A71 CNS involvement are significantly more likely to have symptoms of ADHD than matched healthy controls. Specifically, these children previously infected with EV-A71 have higher scores than matched controls on teacher- and mother-rated scales of inattention, hyperactivity-impulsivity, oppositional symptoms, and ADHD-index. The rate of elevated ADHD-related symptoms among children with EV-A71 CNS infection is 20% while that rate among matched controls is only 3% (*p* < 0.001). They also have more internalizing problems (*p* = 0.003) [34]. Their verbal and performance IQs as well as verbal comprehension indices are significantly inversely correlated with symptoms of inattention, hyperactivity-impulsivity, and ADHD-index scores [34]. The pathogenesis is unclear, and we are undergoing a fMRI study to delineate the possible mechanism.

## Conclusion

In recent 20 years, EV-A71 has become one of the most important pediatric infectious diseases, particularly in the Asia-Pacific region. Although a stage-based management has improved the case-fatality rate of severe EV-A71 cases, long-term sequelae are still of great concern. As identified in follow-up studies of patients with EV-A71 CNS infection, some still have hypoventilation and need tracheostomy with ventilator support. Some infected children have sequelae of dysphagia and need nasogastric tube or gastrostomy feeding. Others have limb weakness/astrophy, cerebellar dysfuction, neurodevelopmental delay, lower cognition, or higher ADHD symptoms. Greater severity of CNS involvement, neuronal damage, hypoxia and younger age of onset may be related to their long term sequelae.

## Data Availability

Not applicable.
